# The precision relationships between eight GWAS-identified genetic variants and breast cancer in a Chinese population

**DOI:** 10.18632/oncotarget.12255

**Published:** 2016-09-26

**Authors:** Yazhen Chen, Fangmeng Fu, Yuxiang Lin, Lin Qiu, Minjun Lu, Jiantang Zhang, Wei Qiu, Peidong Yang, Na Wu, Meng Huang, Chuan Wang

**Affiliations:** ^1^ Department of General Surgery, Fujian Medical University Union Hospital, Fuzhou, Fujian Province, 350001, China; ^2^ Fujian Center for Disease Control and Prevention, Fuzhou, Fujian Province, 350001, China

**Keywords:** breast cancer, GWAS, SNP, stratified analysis, gene-environment interaction analysis

## Abstract

Some of the new breast cancer susceptibility loci discovered in recent Genome-wide association studies (GWASs) have not been confirmed in Chinese populations. To determine whether eight novel Single-Nucleotide Polymorphisms (SNPs) have associations with breast cancer risk in women from southeast China, we conducted a case-control study of 1,156 breast cancer patients and 1,256 healthy controls. We first validated that the SNPs rs12922061, rs2290203, and rs2981578 were associated with overall breast cancer risk in southeast Chinese women, with the per-allele OR of 1.209 (95%CI: 1.064-1.372), 1.176 (95%CI: 1.048-1.320), and 0.852 (95%CI: 0.759-0.956), respectively. Rs12922061 and rs2290203 even passed the threshold for Bonferroni correction (*P* value: 0.00625). In stratified analysis, we found another three SNPs were significantly associated within different subgroups. However, after Bonferroni correction (*P* value: 0.000446), there were no statistically significant was observed. In gene-environment interaction analysis, we observed gene-environment interactions played a potential role of in the risk of breast cancer. These findings provide new insight into the associations between the genetic susceptibility and fine classifications of breast cancer. Based on these results, we encourage further large series studies and functional research to confirm these finding.

## INTRODUCTION

Breast cancer is the most common type of cancer that is diagnosed and the most common cause of cancer deaths among females worldwide [1]. In China, the incidence and mortality from breast cancer is rising rapidly [2]. To effectively reduce the incidence and mortality from breast cancer, its etiology must be determined. Genetic predisposition is an important factor associated with breast cancer risk. Linkage and family based studies have identified numerous predisposition factors for breast cancer, including BRCA1, BRCA2, TP53 and PTEN, which are known as high-penetrance breast cancer susceptibility genes [3–6]. However, only about 5% of the sporadic breast cancer risk and less than 25% of the familial risk can be explained by these high-penetrance susceptibility genes because of their low mutation rates [7]. Meanwhile, GWASs have discovered more than 90 independent low-penetrance susceptibility loci that are associated with breast cancer risk [8]. Different from high-penetrance susceptibility genes, these low-penetrance susceptibility loci account for a substantial portion of the sporadic breast cancer risk and approximately 16% of the familial risk [8]. Most of the susceptibility loci were discovered in women from European populations [9]. Due to the linkage disequilibrium (LD) in diverse populations, it still needs to be determined if these SNPs have strong statistical associations with the risk of breast cancer in other populations. In addition, unlike high-penetrance breast cancer susceptibility genes, each low-penetrance susceptibility loci has only a weak association with the risk of breast cancer, and a small effect for increasing breast cancer risk. However, an accumulative effect of multiple alleles may increase the potential of low-penetrance susceptibility loci, contributing to the risk of breast cancer.

Breast cancer is a complex disease which results from both genetic factors and traditional risk factors. There are a number of traditional risk factors that have been reported to be associated with breast cancer, including age, age at menarche, reproductive and menstrual history, Body Mass Index (BMI), alcohol intake, smoking, physical activity, benign breast diseases, oral contraceptives, and hormone therapy [10–13]. Although many traditional risk factors have been incorporated into risk prediction models for breast cancer [14], little is known about how common susceptibility loci interact with traditional risk factors for breast cancer. The study conducted by the Breast Cancer Association Consortium (BCAC) in 2010 failed to validate the effects of common susceptibility loci on the associations of traditional risk factors with breast cancer [15]. Similarly, a comprehensive study performed by the Breast and Prostate Cancer Cohort Consortium was also unable to validate the effects [16]. However, a study by Nickels et al. including 24 studies from BCAC has provided strong evidence to confirm the important role of genetic-environment interactions in the risk of breast cancer [17]. Moreover, in recent years, exposure data have been incorporated into GWASs, and it is imperative to evaluate gene-environment interactions for breast cancer with the goal of better determining breast cancer susceptibility.

In this study, we conducted a case-control study of 1,156 breast cancer patients and 1,256 healthy controls from a southeast Chinese population to investigate the associations between eight novel GWAS-identified independent genetic susceptibility loci and the risk of breast cancer [18–22]. In addition, we performed stratified analysis, including the subgroups of the breast cancer subtypes, to gain more understanding of these variants in breast cancer etiology. Moreover, we evaluated the combined effects of SNPs. Furthermore, a gene-environment interaction analysis was conducted to explore the role of genetic-environment interactions in the risk of breast cancer.

## RESULTS

A total of 1,156 breast cancer patients and 1,126 health controls were selected for this study, and their characteristics are summarized in Table 1. The age of the breast cancer patients (46.7±10.4 years) was appropriately matched with the age of the controls (47.4±10.8 years). The breast cancer patients were more likely to have a low education lever, a lower mean BMI, fewer live births, a shorter period of breast feeding, an earlier age at menarche, a higher incidence of natural premenopausal status, a higher incidence of prior hormone replacement therapy, a higher incidence of previous benign breast disease diagnosis, and a greater frequency of breast cancer family history, compared with health controls (*P*≤0.05). There were no statistical differences in the other risk factors between patients and controls (*P*>0.05). With regard to the ER/PR status of the breast cancer patients, there were 778 (67.3%) ER positive cases and 709 (61.3%) PR positive cases included in this study.

**Table 1:** Characteristics of breast cancer cases and health controls

Characteristics	Controls (n = 1256)(%)	Cases (n = 1156)(%)	P value
Age (year)(mean ± SD)	47.4±10.8	46.7±10.4	0.118
Education level			1.96×10^−36^
Uneducated or primary school	499 (39.7)	756 (65.4)	
High school or more	757 (60.3)	400 (34.6)	
BMI (kg/m^2^)(mean ± SD)	23.0±3.0	22.6±2.6	4.60×10^−5^
Age at first live birth (year)(mean ± SD)	23.5±5.8	23.8±6.3	0.255
Parity			3.88×10^−12^
0-1	534 (42.5)	655 (56.7)	
≥2	722 (57.5)	501 (43.3)	
Breast feeding period (year)(mean ± SD)	17.6±17.9	15.7±13.3	0.005
Breast feeding period (month)			2.3×10^−6^
<6	294 (23.4)	182 (15.7)	
≥6	962 (76.6)	974 (84.3)	
Age at menarche (year)(mean ± SD)^a^	15.4±1.7	15.1±1.7	5.06×10^−4^
Age at menopausal (year)(mean ± SD)	49.9±3.6	49.8±3.8	0.677
Natural menstrual period (year)(mean ± SD)	29.0±7.2	29.1±7.1	0.82
Menopausal status			0.004
Premenopausal	806 (64.2)	755 (65.3)	
Postmenopausal	426 (33.9)	396 (34.3)	
Unnatural menopausal ^b^	24 (1.9)	5 (0.4)	
Prior hormone replacement therapy			4.66×10^−3^
Yes	28 (2.3)	56 (4.8)	
No	1228 (97.7)	1100 (95.2)	
Previous benign breast disease			0.044
Yes	30 (2.4)	44 (3.8)	
No	1226 (97.6)	1112 (96.2)	
Family history of breast cancer			7.77×10^−16^
Yes	12 (0.96)	86 (7.44)	
No	1244 (99.04)	1070 (92.56)	
ER status			
Positive	778 (67.3)		
Negative	378 (32.7)		
PR status			
Positive	709 (61.3)		
Negative	447 (38.7)		
HER-2 status			
Positive	315 (27.2)		
Negative	841 (72.8)		

a Postmenopausal women only.

b Unnatural menopausal includes hysterectomy operation and other status.

Table 2 shows the allele and genotype distribution of the eight SNPs in breast patients and controls and their association with overall breast cancer risk. Among the control group, the genotype for the eight SNPs are in a Hardy-Weinberg equilibrium (*P*>0.05). After adjusting for age, age at menarche, and family history of breast cancer, we found three of the eight SNPs are significantly associated with overall breast cancer risk. The SNP rs12922061 is most strongly associated with breast cancer risk. The per-allele OR is 1.209 (95%CI: 1.064 to 1.372, *P*=0.003) for the rs12922061 T allele, 1.176 (95%CI: 1.048 to 1.320, *P*=0.006) for the rs2290203 G allele, and 0.852 (95%CI: 0.759 to 0.956, *P*=0.007) for the rs2981578 T allele. Rs12922061 and rs2290203 even passed the threshold for Bonferroni correction (*P* value: 0.05/8=0.00625). We had power of 85.14%, 80.24%, and 78.50% to detect an OR of 1.209, 1.176, and 0.852 for rs12922061, rs2290203, and rs2981578 with the frequency of 0.276, 0.482, and 0.450 respectively. In addition, two SNPs (rs10474352 T allele, OR=0.897, 95%CI: 0.800 to 1.006, *P*=0.062 and rs10816625 G allele, OR=1.113, 95%CI: 0.991 to 1.250, *P*=0.071) have a marginally significant association with breast cancer risk. Specifically, rs2981578 and rs10474352 are associated with a decreased risk, which is contrary to the previous reports [18, 19]. We found that the minor and major alleles of our study and of previous reports are switched, indicating that the difference of the associations is due to different minor alleles among different ethnicities. Additionally, environmental risk factors and breast cancer subtypes must also be considered as possible causes of the difference. No significant association was observed for rs2296067, rs4951011, and rs9693444. However, the statistical power for the five negative loci is <70%. Therefore, some of the null findings may be false negatives.

**Table 2:** Logistic regression analyses on associations between eight SNPs and the risk of breast cancer

SNP	Genes in/near region	Allele^a^	Cases^b^ (n=1156)	Controls^b^ (n=1256)	MAF^c^	HWE	Co-dominant model				Additive model	
							OR_het_^d^ (95%CI)	P_het_^d^	OR_hom_^d^ (95%CI)	P_hom_^d^	OR^d^ (95%CI)	P^d^
rs12922061	CASC16	C/T	534/514/108	659/500/97	0.316/0.276	0.987	1.274 (1.075, 1.511)	0.005*	1.352 (0.998, 1.831)	0.051	1.209 (1.064, 1.372)	0.003*
rs2290203	PRC1	A/G	267/570/319	333/634/289	0.522/0.482	0.929	1.143 (0.935, 1.396)	0.192	1.382 (1.097, 1.740)	0.006*	1.176 (1.048, 1.320)	0.006*
rs2981578	FGFR2	C/T	405/544/207	379/623/254	0.414/0.450	0.998	0.815 (0.678, 0.979)	0.029	0.736 (0.582, 0.932)	0.011	0.852 (0.759, 0.956)	0.007
rs10474352	ARRDC3	C/T	363/569/224	367/602/287	0.440/0.468	0.414	0.950 (0.788, 1.147)	0.596	0.795 (0.632, 1.002)	0.052	0.897 (0.800, 1.006)	0.062
rs10816625	LOC105376214	A/G	349/579/228	431/607/218	0.448/0.415	0.986	1.148 (0.956, 1.380)	0.140	1.226 (0.967, 1.554)	0.092	1.113 (0.991, 1.250)	0.071
rs2296067	KDM4C	G/A	400/570/186	429/614/213	0.407/0.414	0.965	0.988 (0.825, 1.183)	0.896	0.926 (0.726, 1.180)	0.533	0.967 (0.860, 1.087)	0.574
rs9693444	DUSP4-MIR3148	C/A	564/479/113	608/535/113	0.305/0.303	0.955	0.967 (0.815, 1.148)	0.702	1.084 (0.811, 1.447)	0.586	1.012 (0.893, 1.146)	0.854
rs4951011	ZC3H11A	A/G	523/510/123	585/529/142	0.326/0.324	0.404	1.103 (0.928, 1.310)	0.266	0.974 (0.741, 1.281)	0.851	1.025 (0.907, 1.157)	0.694

a Major/minor allele.

b Major homozygote/heterozygote/rare homozygote between cases and controls.

c Minor allele frequency (MAF) among cases/controls.

d Logistic regression with adjustment for age, age at menarche and family history of breast cancer. *P*≤0.00625.

The results of stratified analysis are displayed in Table 3 to Table 6. Regardless of BMI, age at menarche, and the length of the breast feeding period, rs12922061 can increase the risk of breast cancer, and a significant association is observed in women of older age, higher education, premenopausal status, more years of menstruation, younger age at first live birth, more live births, without family history of breast cancer, ER positive, HR positive, and Luminal or HER-2 overexpression type. Subsequently, heterogeneity analysis show that there is heterogeneity between the breast subtypes (*P*=0.031). However, for Luminal or HER-2 overexpression type cases, rs12922061 have a more significant effect (*P*=0.001 and *P=*0.011, respectively). Meanwhile, among women of a younger age, with lower BMI, lower education, a younger age at menarche, postmenopausal status, older age at menopause, more menstruation years, older age at first live birth, longer period of breast feeding, ER positive, HR positive, and with Luminal or HER-2 overexpression type, the association for rs2290203 is significant, regardless of the family history of breast cancer. Moreover, regardless of the family history of breast cancer, rs2981578 shows a protective effect in women of a younger age, with lower BMI, higher education, younger age at menarche, premenopausal status, fewer menstruation years, younger age at first live birth, fewer live births, shorter period of breast feeding, ER positive, HR positive, and Luminal type or HER-2 overexpression type. After heterogeneity analysis, we observed that there are heterogeneities between the two subgroups in age and BMI (*P*=0.036 and *P=*0.032, respectively). Nevertheless, rs2981578 presents a more significant protective effect in the group with a younger age and the group with a lower BMI (*P*=0.002 and *P*=4.53×10^−4^, respectively).

**Table 3:** Stratified analyses between rs12922061, rs2290203, rs2981578, and rs10474352 and breast cancer risk

Characteristics	rs12922061		P^b^	P^c^	rs2290203		P^b^	P^c^	rs2981578		P^b^	P^c^	rs10474352		P^b^	P^c^
	OR(95%CI)	P^a^			OR(95%CI)	P^a^			OR(95%CI)	P^a^			OR(95%CI)	P^a^		
Age (year)			0.231	0.223			0.252	0.227			0.036	0.046			0.734	0.730
≤40	1.073 (0.843, 1.366)	0.565			1.314 (1.059, 1.631)	0.013			0.705 (0.567, 0.877)	0.002			0.869 (0.700, 1.077)	0.199		
>40	1.271 (1.094, 1.476)	0.002			1.124 (0.980, 1.288)	0.095			0.919 (0.802, 1.054)	0.229			0.908 (0.793, 1.039)	0.161		
BMI			0.503	0.468			0.889	0.813			0.032	0.035			0.882	0.879
≤24	1.173 (1.008, 1.365)	0.039			1.185 (1.031, 1.362)	0.017			0.782 (0.682, 0.897)	4.5×10^−4^			0.889 (0.775, 1.021)	0.096		
>24	1.295 (1.022, 1.640)	0.032			1.164 (0.945, 1.435)	0.153			1.066 (0.856, 1.327)	0.569			0.906 (0.737, 1.113)	0.347		
Education level			0.477	0.468			0.362	0.307			0.622	0.592			0.844	0.846
Uneducated or primary school	1.137 (0.951, 1.360)	0.159			1.207 (1.024, 1.423)	0.025			0.855 (0.724, 1.009)	0.064			0.920 (0.783, 1.082)	0.316		
High school or more	1.254 (1.029, 1.527)	0.025			1.078 (0.902, 1.288)	0.412			0.804 (0.673, 0.961)	0.016			0.898 (0.752, 1.073)	0.237		
Age at menarche(year)			0.164	0.872			0.529	0.918			0.646	0.310			0.200	0.131
≤16	1.154 (1.000, 1.331)	0.049			1.161 (1.022, 1.318)	0.022			0.863 (0.759, 0.980)	0.023			0.860 (0.757, 0.977)	0.020		
>16	1.477 (1.113, 1.960)	0.007			1.286 (0.975, 1.695)	0.075			0.804 (0.609, 1.062)	0.125			1.059 (0.813, 1.380)	0.669		
Menopausal status			0.792	0.836			0.441	0.407			0.201	0.200			0.634	0.641
Premenopausal	1.189 (1.012, 1.396)	0.035			1.134 (0.984, 1.308)	0.083			0.806 (0.698, 0.931)	0.003			0.870 (0.755, 1.002)	0.054		
Postmenopausal	1.233 (0.996, 1.527)	0.054			1.252 (1.025, 1.531)	0.028			0.951 (0.780, 1.159)	0.617			0.924 (0.758, 1.127)	0.436		
Age at menopause (year)(postmenopausal women only)			0.978	0.969			0.759	0.751			0.387	0.380			0.296	0.310
<50	1.252 (0.859, 1.825)	0.242			1.179 (0.824, 1.688)	0.368			0.816 (0.572, 1.163)	0.260			1.127 (0.795, 1.598)	0.503		
≥50	1.244 (0.975, 1.587)	0.079			1.261 (1.000, 1.589)	0.050			0.980 (0.780, 1.231)	0.862			0.887 (0.706, 1.113)	0.300		
Years of menstruation(year)			0.065	0.062			0.977	0.995			0.107	0.116			0.464	0.469
<30	1.055 (0.871, 1.278)	0.857			1.175 (0.988, 1.397)	0.067			0.765 (0.643, 0.910)	0.003			0.852 (0.714, 1.016)	0.074		
≥30	1.345 (1.134, 1.595)	0.001			1.171 (1.003, 1.368)	0.045			0.927 (0.793, 1.083)	0.339			0.929 (0.799, 1.079)	0.335		
Age at first live birth (year)			0.857	0.655			0.327	0.746			0.180	0.159			0.455	0.653
≤25	1.205 (1.024, 1.418)	0.024			1.115 (0.959, 1.297)	0.156			0.801 (0.687, 0.933)	0.004			0.928 (0.800, 1.078)	0.329		
>25	1.175 (0.942, 1.465)	0.152			1.264 (1.042, 1.532)	0.017			0.951 (0.787, 1.150)	0.606			0.847 (0.701, 1.023)	0.085		
Parity			0.544	0.583			0.827	0.727			0.799	0.782			0.985	0.923
0-1	1.166 (0.969, 1.403)	0.104			1.173 (0.994, 1.384)	0.059			0.838 (0.709, 0.989)	0.037			0.894 (0.757, 1.056)	0.188		
≥2	1.263 (1.056, 1.509)	0.010			1.204 (1.022, 1.420)	0.027			0.864 (0.733, 1.018)	0.081			0.892 (0.759, 1.047)	0.163		
Breast feeding period (month)			0.373	0.323			0.894	0.881			0.082	0.129			0.531	0.502
<6	1.380 (1.025, 1.858)	0.034			1.152 (0.878, 1.513)	0.307			0.686 (0.515, 0.914)	0.010			0.971 (0.739, 1.276)	0.833		
≥6	1.176 (1.021, 1.355)	0.025			1.176 (1.034, 1.337)	0.013			0.890 (0.784, 1.011)	0.073			0.878 (0.773, 0.996)	0.043		
Family history of breast cancer			0.996	0.994			0.274	0.051			0.239	0.007			0.402	0.171
No	1.209 (1.063, 1.374)	0.004			1.155 (1.028, 1.298)	0.015			0.832 (0.740, 0.935)	0.002			0.888 (0.791, 0.996)	0.042		
Yes	1.206 (0.500, 2.905)	0.677			2.926 (1.145, 7.479)	0.025			3.463 (1.204, 9.961)	0.021			1.687 (0.649, 4.383)	0.283		

a Adjusted for age, age at menarche and family history of breast cancer.

b P for heterogeneity.

c P for interaction. *P*≤0.000446.

**Table 4:** Stratified analyses between rs12922061, rs2290203, rs2981578, and rs10474352 and breast cancer risk by ER status, HR status and subtype

Characteristics	rs12922061		P^b^	rs2290203		P^b^	rs2981578		P^b^	rs10474352		P^b^
	OR(95%CI)	P^a^		OR(95%CI)	P^a^		OR(95%CI)	P^a^		OR(95%CI)	P^a^	
ER status			0.113			0.395			0.874			0.165
Positive	1.283 (1.114, 1.478)	0.001		1.217 (1.069, 1.384)	0.003		0.922 (0.871, 0.977)	0.006		0.947 (0.833, 1.075)	0.399	
Negative	1.067 (0.889, 1.281)	0.485		1.111 (0.940, 1.314)	0.216		0.909 (0.770, 1.074)	0.262		0.818 (0.693, 0.965)	0.017	
HR status			0.087			0.300			0.244			0.095
Positive	1.283 (1.116, 1.475)	4.7*10^−4^		1.221 (1.075, 1.387)	0.002		0.822 (0.723, 0.936)	0.003		0.952 (0.839, 1.079)	0.439	
Negative	1.048 (0.866, 1.267)	0.630		1.091 (0.917, 1.297)	0.325		0.937 (0.790, 1.113)	0.460		0.796 (0.670, 0.946)	0.010	
Subtype			0.031			0.276			0.293			0.555
Luminal	1.294 (1.113, 1.504)	0.001		1.174 (1.023, 1.348)	0.022		0.837 (0.729, 0.961)	0.012		0.936 (0.817, 1.071)	0.336	
HER-2 overexpression	1.285 (1.058, 1.560)	0.011		1.385 (0.152, 1.664)	0.001		0.819 (0.681, 0.985)	0.034		0.886 (0.740, 1.061)	0.189	
Basal-like	0.918 (0.717, 1.177)	0.501		0.976 (0.783, 1.216)	0.828		1.023 (0.823, 1.272)	0.837		0.815 (0.656, 1.014)	0.066	

a Adjusted for age, age at menarche and family history of breast cancer.

b P for heterogeneity. *P*≤0.000446.

**Table 5:** Stratified analyses between rs10816625, rs2296067, rs9693444, and rs4951011 and breast cancer risk

Characteristics	rs10816625		P^b^	P^c^	rs2296067		P^b^	P^c^	rs9693444		P^b^	P^c^	rs4951011		P^b^	P^c^
	OR(95%CI)	P^a^			OR(95%CI)	P^a^			OR(95%CI)	P^a^			OR(95%CI)	P^a^		
Age (year)			0.099	0.125			0.686	0.704			0.911	0.905			0.285	0.259
≤40	0.960 (0.775, 1.188)	0.707			0.930 (0.747, 1.158)	0.517			1.024 (0.813, 1.291)	0.839			1.139 (0.911, 1.424)	0.254		
>40	1.182 (1.029, 1.358)	0.018			0.981 (0.853, 1.127)	0.785			1.008 (0.869, 1.168)	0.919			0.979 (0.847, 1.132)	0.777		
BMI			0.149	0.122			0.618	0.604			0.240	0.185			0.318	0.330
≤24	1.052 (0.915, 1.209)	0.492			0.990 (0.859, 1.140)	0.887			0.956 (0.824, 1.109)	0.551			1.066 (0.922, 1.233)	0.389		
>24	1.282 (1.035, 1.587)	0.023			0.928 (0.750, 1.149)	0.493			1.136 (0.903, 1.431)	0.277			0.933 (0.746, 1.166)	0.540		
Education level			0.450	0.425			0.981	0.960			0.811	0.830			0.283	0.296
Uneducated or primary school	1.194 (1.008, 1.414)	0.040			0.981 (0.828, 1.162)	0.822			1.043 (0.873, 1.245)	0.645			0.960 (0.806, 1.142)	0.641		
High school or more	1.086 (0.910, 1.296)	0.360			0.984 (0.822, 1.177)	0.857			1.010 (0.833, 1.225)	0.916			1.106 (0.918, 1.332)	0.288		
Age at menarche (year)			0.918	0.843			0.107	0.241			0.643	0.187			0.687	0.505
≤16	1.118 (0.984, 1.271)	0.088			0.924 (0.811, 1.053)	0.234			1.027 (0.893, 1.181)	0.709			1.048 (0.914, 1.201)	0.501		
>16	1.100 (0.832, 1.455)	0.504			1.222 (0.927, 1.611)	0.154			0.954 (0.719, 1.265)	0.743			0.984 (0.746, 1.299)	0.910		
Menopausal status			0.779	0.685			0.363	0.343			0.619	0.592			0.246	0.249
Premenopausal	1.094 (0.947, 1.264)	0.221			1.005 (0.869, 1.162)	0.946			1.033 (0.887, 1.204)	0.675			1.083 (0.931, 1.261)	0.300		
Postmenopausal	1.134 (0.927, 1.387)	0.220			0.896 (0.731, 1.098)	0.290			0.966 (0.777, 1.200)	0.752			0.931 (0.755, 1.148)	0.506		
Age at menopause (year)(postmenopausal women only)			0.999	0.865			0.762	0.774			0.818	0.870			0.930	0.926
<50	1.116 (0.781, 1.593)	0.547			0.985 (0.689, 1.407)	0.933			1.029 (0.712, 1.487)	0.879			0.944 (0.670, 1.330)	0.742		
≥50	1.116 (0.886, 1.405)	0.352			0.920 (0.726, 1.167)	0.493			0.975 (0.758, 1.256)	0.847			0.926 (0.723, 1.186)	0.543		
Years of menstruation (year)			0.793	0.751			0.121	0.109			0.203	0.196			0.335	0.335
<30	1.128 (0.947, 1.345)	0.178			1.070 (0.898, 1.274)	0.448			1.111 (0.922, 1.339)	0.267			1.095 (0.913, 1.314)	0.328		
≥30	1.093 (0.936, 1.276)	0.263			0.884 (0.754, 1.036)	0.129			0.941 (0.795, 1.112)	0.475			0.969 (0.823, 1.141)	0.704		
Age at first live birth (year)			0.096	0.017			0.047	0.164			0.818	0.620			0.400	0.846
≤25	1.199 (1.027, 1.400)	0.021			1.050 (0.900, 1.226)	0.532			1.007 (0.855, 1.186)	0.930			0.965 (0.823, 1.130)	0.656		
>25	0.976 (0.809, 1.179)	0.801			0.818 (0.674, 0.994)	0.043			1.039 (0.845, 1.278)	0.718			1.081 (0.881, 1.326)	0.458		
Parity			0.737	0.669			0.391	0.409			1.000	0.986			0.259	0.281
0-1	1.089 (0.922, 1.287)	0.313			0.924 (0.781, 1.093)	0.358			1.009 (0.843, 1.208)	0.924			1.119 (0.936, 1.337)	0.218		
≥2	1.134 (0.961, 1.339)	0.137			1.026 (0.867, 1.213)	0.769			1.009 (0.846, 1.203)	0.919			0.969 (0.817, 1.149)	0.717		
Breast feeding period (month)			0.903	0.827			0.347	0.400			0.060	0.032			0.562	0.624
<6	1.129 (0.852, 1.496)	0.397			0.856 (0.650, 1.126)	0.266			1.340 (1.006, 1.784)	0.046			1.112 (0.835, 1.482)	0.468		
≥6	1.107 (0.973, 1.259)	0.122			0.986 (0.864, 1.124)	0.831			0.946 (0.823, 1.088)	0.437			1.008 (0.880, 1.154)	0.911		
Family history of breast cancer			0.503	0.373			0.156	0.252			0.931	0.927			0.341	0.370
No	1.105 (0.983, 1.243)	0.940			0.976 (0.867, 1.099)	0.686			1.011 (0.892, 1.146)	0.865			1.033 (0.914, 1.168)	0.607		
Yes	1.743 (0.689, 4.414)	0.241			0.556 (0.226, 1.364)	0.199			1.061 (0.422, 2.669)	0.899			0.703 (0.302, 1.637)	0.414		

a Adjusted for age, age at menarche and family history of breast cancer.

b P for heterogeneity.

c P for interaction. *P*≤0.000446.

**Table 6:** Stratified analyses between rs10816625, rs2296067, rs9693444, and rs4951011 and breast cancer risk by ER status, HR status and subtype

Characteristics	rs10816625		P^b^	rs2296067		P^b^	rs9693444		P^b^	rs4951011		P^b^
	OR(95%CI)	P^a^		OR(95%CI)	P^a^		OR(95%CI)	P^a^		OR(95%CI)	P^a^	
ER status			0.099			0.213			0.946			0.452
Positive	1.181 (1.037, 1.346)	0.012		0.918 (0.804, 1.048)	0.206		1.016 (0.884, 1.169)	0.818		0.994 (0.868, 1.139)	0.933	
Negative	0.990 (0.838, 1.170)	0.906		1.055 (0.893, 1.248)	0.529		1.008 (0.842, 1.207)	0.932		1.083 (0.911, 1.288)	0.366	
HR status			0.254			0.530			0.744			0.208
Positive	1.156 (1.016, 1.315)	0.028		0.941 (0.826, 1.072)	0.361		1.025 (0.893, 1.177)	0.722		0.979 (0.856, 1.120)	0.759	
Negative	1.021 (0.859, 1.214)	0.810		1.010 (0.849, 1.202)	0.908		0.986 (0.818, 1.190)	0.887		1.135 (0.949, 1.357)	0.165	
Subtype			0.088			0.486			0.042			0.042
Luminal	1.131 (0.984, 1.299)	0.083		0.921 (0.799, 1.060)	0.250		1.006 (0.866, 1.168)	0.941		0.978 (0.846, 1.130)	0.763	
HER-2 overexpression	1.193 (0.994, 1.431)	0.058		0.967 (0.804, 1.163)	0.718		1.196 (0.988, 1.448)	0.067		0.935 (0.770, 1.135)	0.497	
Basal-like	0.890 (0.711, 1.113)	0.308		1.092 (0.872, 1.367)	0.443		0.804 (0.625, 1.033)	0.088		1.378 (1.104, 1.719)	0.005	

a Adjusted for age, age at menarche and family history of breast cancer.

b P for heterogeneity. *P*≤0.000446.

Although, the other loci are not associated with overall breast cancer risk, there still are significant associations between these loci and different subgroups. The SNP rs10474352 shows a significant protective effect in the women of a younger age at menarche, those with a longer period of breast feeding, those without a family history of breast cancer, who are ER negative, and HR negative. In addition, rs10816625 is significantly associated with the women of an older age, a higher BMI, lower education, younger age at first live birth and who are ER positive and HR positive. Moreover, rs2296067, rs9693444, and rs4951011 are associated with women of an older age at first live birth, shorter period of breast feeding, and Basal-like type, respectively. There are heterogeneities between the subgroup of age at first live birth for rs2296067 (*P*=0.047), between the subgroup of breast subtype for rs9693444 (*P*=0.042), and between the subgroup of breast subtype for rs4951011 (*P*=0.042). Only rs4951011 shows a more significant association with the Basal-like type of breast cancer (*P*=0.005). No heterogeneities were observed in the rest of the subgroups. After Bonferroni correction (*P* value: 0.05/8/14=0.000446), there were no statistically significant was observed in stratified analysis.

As shown in Table 7, we selected the two loci that are significantly associated with overall breast cancer risk to calculate their combined effects (rs12922061-T and rs2290203-G). With no risk allele as the reference, the individual carrying more risk alleles would have a higher OR; 1-2 alleles, OR=1.238, 95%CI: 0.965 to 1.588; 3-4 alleles, OR=1.716, 95%CI: 1.268 to 2.324. It indicated that the combined effect of susceptibility loci would amplify the effect of contributing to the risk of breast cancer (*P* trend =3.97×10^−4^).

**Table 7:** The combined effects of rs12922061 and rs2290203on the association with breast cancer risk

Number of risk alleles^a^	Cases (n=1156) (%)	Controls (n=1256) (%)	OR(95%CI)^b^	Pvalue
0	134 (11.6)	180 (14.3)	1	
1-2	795 (68.8)	894 (71.2)	1.238 (0.965,1.588)	0.094
3-4	227 (19.6)	182 (14.5)	1.716 (1.268,2.324)	4.76×10^−4^
P trend				3.97×10^−4^

a The risk alleles included rs12922061-C and rs2290203-G.

b Adjusted for age, age at menarche and family history of breast cancer.

In the gene-environment interaction analysis, we found that there are interactions between rs2981578 and the age, BMI, and a family history of breast cancer for reducing the risk of breast cancer (*P*=0.046, *P*=0.035, and *P*=0.007, respectively). Additionally, rs10816625 has an interaction with the age at the first live birth for the risk of breast cancer (*P*=0.017). Moreover, there is a significant interaction between rs9693444 and the length of the breast feeding period for breast cancer risk (*P*=0.032) (Table 3 and Table 5).

## DISCUSSION

In the present study, we confirmed that three of the eight SNPs, rs12922061 on 16q12.2, rs2290203 on 15q26.1, and rs2981578 on 10q26.13, are significantly associated with overall breast cancer risk in southeast Chinese women. In addition, rs12922061 and rs2290203 even passed the threshold for Bonferroni correction.

The SNP rs12922061, located in the first intron of *LOC643714*, was identified as a susceptibility variant of breast cancer in a Japanese GWAS [18]. In the present study, we first validated that rs12922061 has a significant association with breast cancer risk in southeast Chinese women, with an allelic OR of 1.209, consistent with the initial GWAS. According to Entrez Nucleotide, it is predicted that the *LOC643714* locus codes for a small mRNA, which could hypothetically be translated into a 55 amino acids protein. However, the specific function of *LOC643714* is still uncertain. A high expression level of *LOC643714* was found in ER positive tumors [23]. In the present study, we observed that rs12922061 has a significant association with the ER positive subgroup, which corresponded with the expression of *LOC643714*. We hypothesize that the SNP rs12922061 may participate in the regulation of the expression of *LOC643714* in ER positive tumors; further analysis is warranted.

The SNP rs2290203 was discovered in an East Asian GWAS, and has been confirmed in European populations [19]. In this study we found that this locus is associated with breast cancer risk in woman from southeast Chinese, and the effect is similar to the initial GWAS (OR=1.176; 95%CI: 1.048 to 1.320). This locus lies in intron 14 of the protein regulator of the cytokinesis 1 (*PRC1*) gene, which encodes the PRC1 protein and is suspected of being strictly regulated in a cancer-specific manner. The PRC1 protein is a mitotic spindle midzone-associated protein and is a substrate of a cyclin-dependent kinase [24]. The *PRC1* gene is down-regulated by p53, whereas in p53 defective cells, it is over-expressed [25]. Also, the expression level of the *PRC1* gene is significantly higher in breast tumor tissue, compared with adjacent normal tissue [19]. A study indicated that the higher expression level of the *PRC1* gene could be a predictor of the poor prognosis for breast cancer patients [26]. However, there is no association reported between rs2290203 and the expression of the *PRC1* gene, but it does relate to the *PCCD1* gene (5,712 bp upstream of rs2290203) [19, 27]. We still don't know the functions of the *PCCD1* gene.

The SNP rs2981578 is located in intron 2 of the fibroblast growth factor receptor 2 (*FGFR2*) gene. In 2007 this gene was reported to be associated with breast cancer in women of European descent [28, 29]. Subsequently, rs2981578 was discovered to be associated with breast cancer in a fine-mapping study of African American populations [30]. This locus has also been identified in an African population [31]. Interestingly, in our study, we found that rs2981578 is associated with a decreased breast cancer risk, which is contrary to the previous reports (OR=0.852; 95%CI: 0.759 to 0.956). This may be due to the diverse genetic background among different ethnicities combined with environmental risk factors and breast cancer subtypes. Additionally, fibroblast growth factor receptor type 2, encoded by the *FGFR2* gene, is a receptor tyrosine kinase, which is an essential part of the signaling pathway of the growth and differentiation for cells in breast tissue [32]. Meanwhile, rs2981578 is reported to cause differential expression of the common and minor haplotypes of the *FGFR2* gene [33].

In postmenopausal women the endogenous estrogen is mainly provided by adipose tissue [34], and it is well demonstrated that estrogen has a significant linear correlation with breast cancer in these women [35]. It was also reported that some polymorphisms in *FGFR2* were associated with breast cancer risk in postmenopausal women [36]. From our results, we believe that rs2981578 is correlated with reducing breast cancer risk in women of a younger age, lower BMI, younger age at menarche, premenopausal status, and fewer menstruation years; particularly with ER positive, HR positive, and Luminal type. The gene-environment interaction analysis also showed that rs2981578 is interacting with BMI. Considering the above results, we speculated that rs2981578 may play an important role in regulating pathways which are related to estrogen. Nevertheless, further functional research is still needed to confirm the relationship of the susceptibility locus and breast cancer.

For rs2296067, rs4951011, and rs9693444, no significant ORs were observed in the present study. However, there were still significant associations between these loci and different subgroups. Moreover, it has been previously reported that rs9693444 is associated with breast cancer risk in Chinese women (*P*=6.44×10^−4^) [37]. We believe that the reason for failing to confirm their previously established role in breast cancer risk is that there are difference LD patterns in difference populations. In addition, environmental risk factors and breast cancer subtypes should also be taken into consideration.

Several limitations need to be taken into consideration in this study. Above all, the sample size in our study is still limiting and that will affect the sensitivity and accuracy of the results. Particularly in the stratified analysis, the incidences of some epidemiological characteristics were not numerous enough and cannot be used to efficiently analyze our data. In addition, as our study was a hospital based case-control study, there was a certain selection bias compared with the general population. The self-reported life-style factors of participation might also have a recall bias. Therefore, in the next few years, we will expand the sample size and perform a large series study to improve the sensitivity and accuracy of the study, aiming to reduce these biases, and better understand the relationship between breast cancer risk and these susceptibility loci.

In conclusion, our study is the first study to validate that rs12922061 on 16q12.2, rs2290203 on 15q26.1, and rs2981578 on 10q26.13 are associated with overall breast cancer risk in southeast Chinese women. The SNPs rs12922061 and rs2290203 even passed the threshold for Bonferroni correction. In addition, the three other SNPs (rs10474352 on 5q14.3, rs10816625 on 9q31.2, and rs4951011 on 1q32.1) are found to have a significant association within different subgroups. Moreover, gene-environment interaction analysis revealed that there are interactions between rs2981578 and the age, BMI, and family history of breast cancer, between rs10816625 and the age at the first live birth, and between rs9693444 and the length of the breast feeding period. These findings may provide new insight into the association between genetic susceptibility and the fine classifications of breast cancer, which would guide clinical therapy in the future. Finally, it is certain that further large series studies and functional research are still warranted.

## MATERIALS AND METHODS

### Study participants

All study participants were genetically unrelated Chinese females from Fujian province. There are 1,166 breast cancer patients and 1,258 healthy controls in this hospital-based case-control study. Patients were randomly enrolled from Fujian Medical University Union Hospital, Fujian, China, between January 2005 and December 2015, and each case was histopathologically confirmed by at least two oncologists. Estrogen receptor (ER) status, progesterone receptor (PR) status and human epidermal growth factor receptor 2 (HER-2) status of patients were evaluated by immunohistochemical analysis. It was considered to be a positive result when the percentage of stained cancer cell nucleus were ≥10%. The rest of the clinicopathological data was obtained from medical records. Healthy controls were selected from people who were undergoing routine health examinations in the same hospital during the corresponding period. Controls were age-matched (±5 years) healthy individuals without breast cancer and other cancers. After written informed consent was obtained, each study participant was personally interviewed face-to-face by trained interviewers to collect information that included demographic data, menstrual, reproductive and breastfeeding history, environmental exposure history, previous benign breast disease history, and their family history of breast cancer. Subsequently, a 3-ml peripheral blood sample was collected. This study was approved by the Ethics Committee of Fujian Medical University Union Hospital.

### SNP selection and genotyping

We selected 23 novel GWAS-identified SNPs by reviewing the available literature between January 2013 and December 2015 [18–22, 38–40]. There were 12 SNPs be excluded because they did not achieved genome-wide significance (*P*≤5.0×10^−8^). The SNP rs13294895 was excluded owing to have an minor allele frequency (MAF) <0.1 in Han Chinese from Beijing from HapMap. The MAF of rs140068132 in Han Chinese from Beijing from HapMap have not been reported, therefore rs140068132 was removed. A further SNP rs3803662 has been incorporated in our foregoing experiments. Thus, the remaining eight SNPs were selected for genotyping, namely 16q12.2-rs12922061 (*CASC16*), 15q26.1-rs2290203 (*PRC1*), 10q26.13-rs2981578 (*FGFR2*), 5q14.3-rs10474352 (*ARRDC3*), 9q31.2-rs10816625 (*LOC105376214*), 9-rs2296067 (*KDM4C*), 8p12- rs9693444 (*DUSP4-MIR3148*), and 1q32.1-rs4951011 (*ZC3H11A*).

Genomic DNA was extracted from leukocytes from EDTA anti-coagulated whole blood using the Whole-Blood DNA Extraction Kit (Bioteke, Beijing, China), according to the manufacturer's protocol. The concentration of the DNA samples was quantified with an Epoch Microplate Spectrophotometer (BioTek Instruments, Winooski, VT, USA), and quality of DNA samples was determined by agarose gel electrophoresis. Qualified DNA samples were genotyped by SNPscan, which is a high-throughput SNPs genotyping technology (Genesky Biotechnologies Inc., Shanghai, China). Finally, the raw date was analyzed by the GeneMapper 4.0 Software (Applied Biosystems, Foster City, CA). A five percent sample of both the cases and controls were randomly selected as blinded duplicates for quality assessment purposes and 100% agreement was obtained. Due to DNA quality or quantity, genotyping of ten cases (0.86%) and two controls (0.16%) failed. The call rate for per-SNP was 99.5%. After removing all data from these 12 participants, there were 1,156 cases and 1,256 controls in the final analyses.

### Statistical analyses

Differences in demographic characteristics, risk factors and frequencies of alleles and genotypes between cases and controls were evaluated by t-test, for continuous variables, or χ^2^ tests, for categorical variables. Genotype data of control samples were evaluated for consistency with the Hardy-Weinberg equilibrium (HWE) by a goodness-of-fit χ^2^ test. The associations between SNPs and the risk of breast cancer were assessed by computing odds ratios (ORs) and 95% confidence interval (CIs) using conditional logistic regression models (co-dominant model and additive model) with adjustment for potential confounders such as age, age at menarche, and family history of breast cancer. The power of the study was carried out by using the Quanto, version 1.2.4, with the disease risk in the Chinese population was 268.6 per 100000. The date was then stratified into fourteen subgroups (Table 3 to Table 6). Subsequently, we used the χ^2^-based Q-test to estimate the heterogeneity of associations within subgroup. Moreover, we categorized all cases and controls into five groups based on the number of risk alleles they carried (from 0 to 4, with 0 risk alleles used as the reference), and assessed the cumulative effect of multiple genetic risk variants by calculating OR and 95%CI with adjustment for potential confounders as described above. Furthermore, the genetic-environment multiplicative interaction analysis was applied to explore the interactions between susceptibility loci and traditional risk factors, and it was performed by a multinomial logistic regression model. All of the statistical analyses were two-sided and a *P* value equal to or less than 0.05 was taken as the significance level. The Bonferroni correction was adopted to correct multiple comparisons. Analyses were carried out by using the Statistical Package for the Social Sciences (SPSS, version 18.0)
